# The American Medical Association

**Published:** 1882-03-20

**Authors:** 


### *THE AMERICAN MEDICAL ASSOCIATION*

Will assemble the present year at St. Paul, Minnesota, on the first Tuesday in June, next. A large representation is anticipated, especially from the South.

---

